# Extensive multivariate dataset characterizing bacterial community diversity and *Campylobacter* contamination level in a large number of conventional broilers carcasses after air chilling and refrigerated storage

**DOI:** 10.1016/j.dib.2024.110858

**Published:** 2024-08-20

**Authors:** Sophie Hautefeuille, Raouf Tareb, Agnès Bouju-Albert, Boris Misery, Béatrice Laroche, Sandrine Guillou, Nabila Haddad

**Affiliations:** a Oniris, INRAE, SECALIM, 44300 Nantes, France; b Université Paris-Saclay, INRAE, MaIAGE, Jouy-en-Josas, France

**Keywords:** Chicken meat, Microbiota, Slaughterhouse, Metagenetic, Pathogen, Food safety

## Abstract

The article provides extensive data on the diversity of bacterial communities collected on 480 conventional broiler carcasses from a French slaughterhouse between October 2022 and January 2023. Half of the carcasses were collected from the first slaughter batches and the other half from the later batches to highlight possible microbial cross-contamination between carcasses along the slaughter chain. *Campylobacter* and the Total Viable Count were quantified on agar plates before and after 7 days of refrigerated storage to estimate the contamination level of broiler carcasses. These data on *Campylobacter* quantification allow us to assess the prevalence and concentration of this pathogen on chicken meat in France. Broiler bacterial communities were sequenced and characterized before and after storage, offering an understanding of broiler carcass microbial diversity and ecology on large-scale data along the food chain. Amplicons from the region V3-V4 of 16S rRNA were sequenced with Illumina MiSeq technology. Raw sequencing data were deposited in the ANR-21-CE21–0008 ESCAPE project, and the sample accession numbers for the rRNA 16S sequencing range from J017_lib692622_cleaned_R1 to J0176_lib692782_cleaned_R2 for the samples before storage and from J717_lib692783_cleaned_R1 to J7176_lib692988_cleaned_R2 for the samples after storage.

Specifications Table

**Table:** 

Subject	Food microbiology
Specific subject area	Microbial ecology of the broiler carcass bacterial communities and quantification of the foodborne pathogen *Campylobacter* contamination level after industrial production
Type of data	Table, Figure, Raw sequencing data
Data collection	480 conventional broiler carcasses were collected after the chilling step with sterile sampling bags, stored at 4 °C, and transported in large coolers. Each carcass was cut in half and weighed. One-half of the carcass was packaged with plastic film on a tray and stored at 4 °C for 7 days. Each carcass half was rinsed in 2 L full-page filter Stomacher bags of Ø 280 µm (Interscience, France) with 700 ml of buffered peptone water (Laboratoire Humeau, France). The bags were kneaded manually for 15±5 s and shaken at 150 rpm (Infors HT Minitron) for five minutes at ambient temperature. The rinses from three carcass halves were pooled and mixed with a magnetic bar for 15±5 s. *Campylobacter* was quantified on CASA agars (Biomérieux, France) by plating 1 mL of 10-fold concentrated pooled rinses on three plates and tenfold serial dilutions of the concentrate on three plates. Agar plates were incubated under microaerobic conditions (5 % O2, 10 % CO2, and 85 % N2) at 42 °C for 48 h. Dark red colonies were counted, and concentrations were calculated. To determine the Total Viable Count, 100 µL of each serially diluted pooled rinse (10–1, 10–2, 10–3, 10–4, 10–5) was plated onto PCA plates (VWR, France). The agar plates were then incubated at 30 °C for 48 h, and the concentrations were determined according to the NF EN ISO 7218 norm. Total Viable Count by plating 100 µL of each serially diluted pooled rinse (10–1, 10–2, 10–3, 10–4, 10–5) onto the surface of PCA plates (VWR, France). Agar plates were incubated at 30 °C for 48 h, and concentrations determined using NF EN ISO 7218 norm. Pellets were obtained from successive centrifugations of the pooled rinses. DNA was extracted using the PowerFood Microbial Kit (Qiagen, France). Extracted samples were quality checked with Implen Nano Spectrophotometer® N50 (Implen) and sequenced through the sequencing of the 16S rRNA V3-V4 hypervariable region according to the manufacturer's instructions with Illumina MiSeq by Eurofins Genomics.
Data source location	The slaughterhouse where the carcasses were collected requested anonymity. The carcasses were analyzed in our unit: SECALIM, Oniris/INRAE, Nantes, France, GPS coordinates: Latitude: 47.289464 | Longitude: −1.52275.
Data accessibility	Repository name: Raw sequencing data and metadata are available in the Institutional INRAE data repository. Data identification number: Raw sequencing data are available at: https://doi.org/10.57745/WUII9H. Metadata are available as supplementary data (Excel spreadsheet) at the : https://doi.org/10.57745/ETSMIM Direct URL to data: URL for the raw sequencing data and metadata respectively: https://entrepot.recherche.data.gouv.fr/dataset.xhtml?persistentId=doi:10.57745/WUII9H and https://entrepot.recherche.data.gouv.fr/dataset.xhtml?persistentId=doi:10.57745/ETSMIM

## 1 Value of the Data

• The data describe the diversity/composition of the bacterial communities in broiler carcasses before storage (after chilling step) and after 7 days of refrigerated storage (under air).
• The availability of high-resolution sequencing data can provide resource for exploring the interactions between different microbial species.
• Species characterized in sequencing data can be used for other purposes, such as identifying other emerging pathogens or spoilage biomarkers.
• Quantification of *Campylobacter* on broiler carcasses before storage (after chilling step) and after 7 days of refrigerated storage (under air) can be used for risk assessments.
• The data can establish a correlation between the level of contamination of *Campylobacter* and the diversity/composition of the bacterial communities in broiler carcasses before and after storage.

## 2 Background

*Campylobacter* has been Europe's leading cause of foodborne zoonosis for several years, with poultry as the leading risk factor. Controlling its contamination in poultry meat is a major public health concern due to high number of campylobacteriosis cases. Throughout the food chain, various factors influence the bacterial flora composition on chicken carcasses and the level of *Campylobacter* contamination. In this study, we seek to understand how storage, different slaughter batches, and other factors, affect the composition and diversity of the bacterial flora, as well as the level of *Campylobacter* contamination. Moreover, while studies have examined *Campylobacter*'s survival under various stresses in the food chain, none have considered its interaction with microbiota. Given that *Campylobacter* coexists with diverse microbial communities, we also seek to characterize how the composition of broiler carcasse microbiota affects its contamination levels. Investigating these dynamics, including the interactions between the bacterial flora and *Campylobacter*, is essential for developing effective strategies to control contamination and ensure food safety.

## 3 Data Description

This dataset describes the microbial diversity of 480 conventional broiler chicken samples collected in ten different flocks between 2022 and 2023 in a French slaughterhouse. Half of the carcasses were collected from the first slaughter batches (FSB), and the other half from the later slaughter batches (LSB), as indicated in Table S01, which describes the samples and their metadata. The carcasses were cut in half to be analyzed before and after storage (Table S01, Storage = D0 or D7, respectively). The half-carcasses underwent rinsing before being pooled in groups of three to form one sample, to avoid large variability between samples, resulting in 320 samples. The 320 samples were sequenced by amplifying the V3-V4 region of the 16S rRNA and deposited in the ANR-21-CE21–0008 (ESCAPE) project in the INRAE Institutional Data Repository under this DOI: https://doi.org/10.57745/WUII9H. Sample accession numbers for the 16S rRNA sequencing, including forward (R1) and reverse (R2) reads, range from J017_lib692622_cleaned_R1 to J0176_lib692782_cleaned_R2 for the samples before storage and from J717_lib692783_cleaned_R1 to J7176_lib692988_cleaned_R2 for the samples after storage. The sequencing data analyzed before and after storage, including tables for ASV abundance, ASV taxonomic assignment, and sample metadata for use, are available in Tables S03 and S04, respectively. In parallel, we quantified *Campylobacter* and Total Viable Count (TVC) on agar plates before and after storage and are described in Table S02. All supplementary tables (Table 1) are available and can be downloaded under this DOI: https://doi.org/10.57745/ETSMIM.

**Table 1:** Supplementary tables comprising the dataset are available as spreadsheets at https://doi.org/10.57745/ETSMIM.

Table S01	Sample nomenclature and corresponding metadata
Table S02	Agar-plate quantification of *Campylobacter* and the Total Viable Count
Table S03	Analysis of bacterial diversity through sequencing of the 16S rRNA V3-V4 region amplicons before storage, including tables for ASV abundance, ASV taxonomic assignment, and sample metadata for use with the phyloseq R package.
Table S04	Analysis of bacterial diversity through sequencing of the 16S rRNA V3-V4 region amplicons after storage, including tables for ASV abundance, ASV taxonomic assignment, and sample metadata for use with the phyloseq R package.
Table S05	This document provides an overview of the dataset, sequencing methods, analyzed data, and metadata associated with the study of microbial diversity in broiler chicken samples.

## 4 Experimental Design, Materials And Methods

### 4.1 Sampling and microbiological analysis

**Fig. 1:**
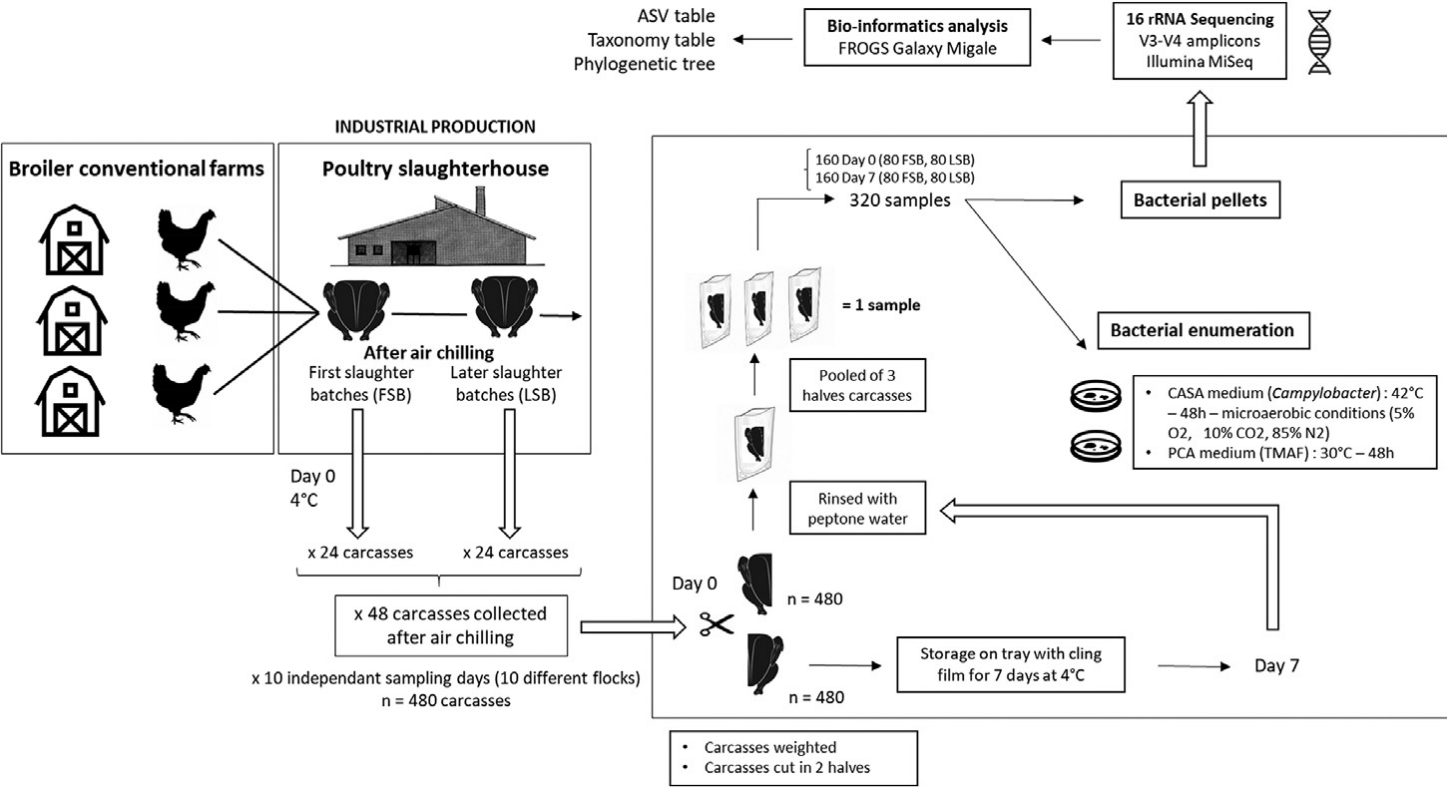
Large-scale methodology for analyzing whole broiler carcasses.

480 conventional broiler carcasses were collected over ten non-consecutive slaughtering days, spanning from September 2022 to January 2023, in a slaughterhouse in northwestern France. To capture variability associated with the time of day during slaughtering, half of the carcasses were collected from the first batches of the day and the other half from the later batches. These carcasses were extracted from the slaughter line right after chilling, placed in sterile sampling bags, and stored at 4 °C (Fig. 1). The carcasses were weighed, identified, and then halved on the day of sampling. One portion of each carcass (“D0” samples) was stored overnight at 4 °C and analyzed, while the other half was packed with plastic film on a tray and stored at 4 °C for 7 days before analysis (“D7” samples), allowing for the assessment of storage effects.

### 4.2 Microbiological analysis

Microbiological analysis was performed the day after slaughter and after 7 days of storage at 4 °C, on the pooled rinses of the three corresponding half-carcasses. First, each half-carcass was rinsed in 2 L full-page filter Stomacher bags from Interscience (Ø 280 µm) with 700 ml of buffered peptone water. The bags were kneaded manually for 15±5 s and shaken at 150 rpm for five minutes at ambient temperature. The rinses from three carcass halves were pooled and mixed with a magnetic bar for 15±5 s.

Two quantitative analyses were carried out on each of the pooled rinses to determine contamination levels of the 320 samples, representing 960 half carcasses pooled in groups of three under the same condition.

a. *Campylobacter* contamination level

*Campylobacter* detection and quantification were carried out using semi-selective CASA agars. To upper the quantification limit of *Campylobacter* to 0.7 log CFU/ml of carcass rinse, we concentrated 10-fold the pooled rinses through centrifugation. Enumeration of *Campylobacter* involved plating 1 mL of the concentrate on three CASA plates. Additionally, tenfold serial dilutions of the concentrate in peptone water were prepared and plated onto one CASA plate. Following incubation under microaerobic conditions at 42 °C for 48 h, dark red colonies were counted, and concentrations were calculated.

b. total viable count

For the quantification of Total Viable Count (TVC), 100 µL of each serially diluted pooled rinse (10^−1^, 10^−2^, 10^−3^, 10^−4^, 10^−5^) was inoculated onto the surface of PCA plates. Agar plates were incubated at 30 °C for 48 h, and concentrations were determined using NF EN ISO 7218 norm.

### 4.3 Bacterial pellet and DNA extraction

To extract DNA, we obtained bacterial pellets from each rinse as described below. 160 ml of rinses were centrifuged at 600 g for 5 min, to remove debris. 40 ml of supernatant was then centrifuged at 10 000 g for 10 min. The pellets were resuspended with 1 ml peptone water and centrifuged at 10 000 g for 5 min. Bacterial pellets were stored at −20 °C prior to DNA extraction. DNA extractions from bacterial pellets were performed using the PowerFood Microbial Kit (Qiagen), according to the manufacturer's recommendations with some modifications as follows. Briefly, the pellets were resuspended with 450 µl of Solution MBL, then agitated in PowerBead Tubes during 30 s at 6 m.s-1 with the FastPrep-24™ 5 g (MP Biomedicals). The tubes were centrifuged at 13,000 x g for 1 min and supernatants were mixed with 100 µl of IRS Solution before 30 min of incubation in crushed ice, instead of 5 min. Supernatants were collected after 13,000 x g for 1 min, mixed with 900 µl of MRSolution, and loaded onto MB Spin Column. We added 650 µl of PW Solution and 650 µl of Ethanol successively to the membranes, before drying it at 50 °C for 10 min before elution to avoid contaminants. DNA elution was performed with 100 µl of ultrapure water, instead of EB Solution. Each final extract contained 100 µl of DNA extract in ultrapure water, and was stored at −20 °C until sequencing. DNA concentration and quality were assessed using the Implen Nano Spectrophotometer® N50 of the Secalim unit. Additionally, Tuf qPCR was conducted on selected samples to monitor the presence of amplification inhibitors by quantitative PCR kit (Takara Bio.), according to the manufacturer's instuction.

### 4.4 16 rRNA sequencing and bioinformatics analysis

20 µl (DNA mean concentration = 299 µg/µl) of each extracted sample were sent to Eurofins Genomics in Germany. The high-throughput sequencing of the V3-V4 hypervariable region of the bacterial 16S rRNA was performed using an Illumina MiSeq platform according to the manufacturer's instructions. Amplicons were generated using a two-step PCR protocol. Briefly, the V3-V4 regions were PCR-amplified using template specific primers (forward: TACGGGAGGCAGCAG [1], reverse: CCAGGGTATCTAATCC [2]). The amplicons were cleaned up and set up for the PCR index, with specific primers directed to universal overhangs. Final amplicons libraries were cleaned up, quantified and pooled equimolar. The resulting final library pool was quantified and sequenced using the 2 × 300 bp paired-end reads chemistry. A first preprocess was realized by the sequencing platform Eurofins. Quality of raw sequencing was first checked and filtered to retain only high quality bases by performing adapter trimming using Cutadapt software [3], quality filtering using Phred Quality Scores (Q30 > 75 %) and per-read quality pruning. Primers removed paired-end reads were merged using the software FLASH [4], with a minimum overlap size of 10 bp to reduce false positive merges. Bioinformatics analysis was carried out using FROGS (Find Rapidly OTUs with Galaxy Solution) on the Galaxy Migale platform [5]. A second preprocess (Galaxy Version 4.1.0+galaxy1) was performed for the merging, denoising, and dereplication. The maximum rate of mismatches in the overlap region was set at 0.1, and Vsearch was used to merge paired-end reads. Then, we used Clustering swarm on the dereplicate sequences to do single-linkage clustering on sequences with an aggregation distance of 1. Chimeras were removed in each sample with Vsearch. Clusters were filtered with a minimal prevalence of 80 and a minimum proportion of sequence abundance of 0.0005, and ASV affiliated with Blast using 16S SILVA Pintail100 138.1 [6]. The Affiliation explorer of shiny migale [7] allowed us to select the multi-affiliations manually if several species were characterized in the same ASV. Three abundance tables (D0, D7 and D0+D7 samples) were made to not bias the analysis towards extremely abundant taxa between D0 and D7 samples. In total, we kept 318 samples, after subtracting two non-amplifiable samples.

## Limitations

None.

## Ethics Statement

All the authors have read and followed the ethical requirements for publication in Data in Brief and confirmed that the current work does not involve human subjects, animal experiments, or any data collected from social media platforms.

## Data Availability

Replication Data for "Extensive multivariate dataset characterizing bacterial community diversity and Campylobacter contamination level in a large number of conventional broilers carcasses after air c (Original data) (Data Gouv). 16S RNA metabarcoding dataset from microbiota of conventionnal broiler carcass rinses (Original data) (Data Gouv).
